# Genetic Diversity and Evolution of Satellite RNAs Associated with the Bamboo Mosaic Virus

**DOI:** 10.1371/journal.pone.0108015

**Published:** 2014-10-02

**Authors:** Ing-Nang Wang, Chung-Chi Hu, Ching-Wei Lee, Sih-Min Yen, Wen-Bing Yeh, Yau-Heiu Hsu, Na-Sheng Lin

**Affiliations:** 1 Institute of Plant and Microbial Biology, Academia Sinica, Taipei, Taiwan, Republic of China; 2 Department of Biological Sciences, University at Albany, Albany, New York, United States of America; 3 Graduate Institute of Biotechnology, National Chung Hsing University, Taichung, Taiwan, Republic of China; 4 Department of Entomology, National Chung Hsin University, Taichung, Taiwan, Republic of China; University of California, Riverside, United States of America

## Abstract

Satellite RNAs (satRNAs) are subviral agents that depend on cognate helper viruses for genome replication and encapsidation. Their negative impacts on helper viruses have been exploited to control plant viral diseases. SatBaMV is a commonly found satRNA associated with *Bamboo mosaic virus* (BaMV) that infects diverse bamboo species in the field. To investigate the genetic diversity and evolution of satRNAs, we examined seven satBaMV populations derived from five bamboo species and cultivars from Taiwan, China, and India and one from the greenhouse. We found 3 distinct clades among the seven populations. Clade I is consisted of all satBaMV isolates, except for those from *Dendrocalamus latiflorus* in Taiwan and *Bambusa vulgaris* in India, which belong to Clades II and III, respectively. Interestingly, nucleotide diversity was lower for Clade I than II and III. However, the nucleotide diversity did not seem to depend on bamboo species or geographic location. Our population genetic analyses revealed the presence of excessive low-frequency polymorphic sites, which suggests that the satBaMV population was under purifying selection and/or population expansion. Further analysis of *P20*, the only satBaMV gene that encodes a non-structural protein involved in the long-distance movement of satBaMV, showed evidence of purifying selection. Taken together, our results suggest that purifying selection against defective P20 protein is responsible at least in part for the evolution of the satBaMV genome.

## Introduction

One of the most striking and under-appreciated differences between plant and animal viruses is the predominant association of satellite RNAs (satRNAs) with the plant viruses [1]–[3]. The genomes of satRNAs are small, usually <1,500 nucleotides (nt) [1]. Their sequences do not reveal any recognizable open reading frame (ORF) or show only a single ORF encoding a small protein. Because of their small genome size, satRNAs do not encode their own RNA-dependent RNA polymerases (RdRp) or coat proteins (CPs), so they depend on their cognate helper viruses for genome replication and encapsidation [1]–[5]. Therefore, satRNAs are commonly seen as molecular parasites [3], [6] engaging in a zero-sum game by exploiting vital functions provided by their helper viruses.

One manifestation of this antagonistic interaction between satRNAs and their helper viruses is the attenuation of disease symptoms, presumably as a result of reduced viral titers in the host plants. Engineered satRNAs exploit this interfering capacity as antiviral agents for crop protection [7]–[10]. However, empirical studies have shown that the effects of satRNAs on their helper viruses can run the full gamut of all possible interactions, from antagonistic to beneficial [2], [11], resulting in a wide range of disease symptoms [12]. One contributing factor to such variable interactions may be the fascinating interplay among three parties: the satRNA, the helper virus, and the host defense mechanisms, most notably the RNA silencing pathway [1].

Besides the satRNAs associated with *Cucumber mosaic virus* (CMV) and *Turnip crinkle virus* [13], satRNAs associated with the *Bamboo mosaic virus* (satBaMVs) are another of the intensively studied model systems. BaMV was first discovered in Brazil [14]. However, the most extensively studied isolates were first isolated from infected green bamboos (*Bambusa oldhamii* Munro) in Taiwan [15]. BaMV is a plant potexvirus with a filamentous, flexuous morphology of approximately 490 by 15 nm. The BaMV-O genome is a positive-stranded RNA of 6366 nt, with 5 ORFs [16]. The genome for satBaMV is 836 nt (without the poly-A tail) and encodes a single gene product of 183 amino acid residues [17] with similarity to the CP encoded by the satellite virus of *Panicum mosaic virus* [18]. The encoded 20-kDa protein is not required for satBaMV replication [19], but it does preferentially bind to the satBaMV RNA [20], presumably participating in the regulation of systemic movement of satBaMV in the host plant [19], [21], [22].

Because the replication of satBaMV depends on the RdRp encoded by BaMV, both the 5′ [23], [24] and 3′ [25] untranslated regions (UTRs) of the satBaMV and BaMV RNAs, not surprisingly, share structural similarities. Therefore, competition for the limited intracellular resources (*e.g.*, RdRp) can lead to an antagonistic interaction between these two entities, thus resulting in reduced BaMV titers and consequently attenuated symptoms [26]–[28].

However, not all satBaMV isolates behave antagonistically toward their helper virus. For example, the satBaMV isolate BSL6, first isolated from Ma bamboo (*Dendrocalamus latiflorus* Munro) [29], strongly interferes with BaMV replication [30], whereas BSF4, first isolated from *B. vulgaris* [17], does not seem to have a significant effect on BaMV [19], [30]. Detailed genetic studies showed that various mutations at the 5′ apical hairpin stem loop (AHSL) region affect the ability of satBaMV to interfere with the replication of its helper virus [27], [28]. A single nucleotide change of U to C at position 82 within the AHSL region of BSF4 can turn the non-interfering BSF4 into an interfering satRNA [28]. Therefore, the interfering phenotype is quite malleable and, presumably, is easily accessible via mutation.

In contrast to many detailed molecular studies, evolutionary and population studies of satRNAs are not as broad or as in-depth. The best-studied example is the various satRNAs associated with CMV [31]–[34]. At present, no systematic population study has investigated satBaMV, except for the previous surveys of field samples of bamboo species in Taiwan [23], [29].

In this study, we collected, sequenced, and analyzed by phylogenetic and population genetics analyses of satBaMV isolates from five bamboo species and cultivars in three main geographic locations in Asia. We identified three distinct satBaMV clades and inferred the evolutionary forces responsible for the observed variations in our collection of satBaMV sequences.

## Materials and Methods

### Sample collection and preparation

**Figure 1:**
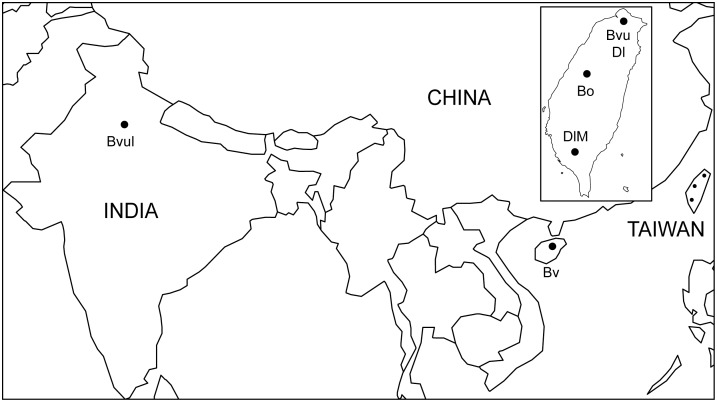
Map showing the geographic locations of collected bamboo samples. Inset shows an enlarged map of Taiwan for clarity. The name designations for each satBaMV populations are: BvuI, from *B. vulgaris* Schrad. *ex* J. C. Wendl. at Delhi, India; Bv, from *B. ventricosa* McClure at Hainan Island, China; Bvu, from *Bambusa vulgaris* Schrad. *ex* J. C. Wendl. at Taipei; Dl, from *Dendrocalamus latiflorus* Munro at Taipei; Bo, from *B. oldhamii* Munro at Taichung, Taiwan; and DlM, from *D. latiflorus* Munro cv. Mei-nung W. C. Lin at Pingtung, Taiwan.

Leaf samples showing visible mosaic symptoms from various bamboo species were collected from locations in northern (*B, vulgaris* Schrad. *ex* J. C. Wendl. and *D. latiflorus* Munro at the Taipei Botanical Garden; GPS coordinates: 24.91571, 121.67394), central (*B. oldhamii* Munro at Chung Hsing Lake of National Chung Hsing University at Taichung; GPS coordinates: 24.23321, 120.94174) and southern (*D. latiflorus* Munro cv. Mei-nung W. C. Lin at Pingtung; GPS coordinates: 22.67611, 120.49417) Taiwan in 2006; Hainan Island of China (*B. ventricosa* McClure; GPS coordinates: 19.00000, 109.50000) in 2007; and Delhi, India (*B. vulgaris* Schrad. *ex* J. C. Wendl.; GPS coordinates: 28.63531, 77.22496) in 2009. The presence of BaMV and satBaMV was checked by dot or northern blot analysis with BaMV and satBaMV-specific probes, respectively [19], [35]. To ensure a fair representation of the satBaMV population, leaves from several individual bamboo plants in a given location were combined for total RNA extraction (see below). Figure 1 shows the locations of leaf samples and name designations for satBaMV populations.

To establish a baseline evolution in the greenhouse condition, *Nicotiana benthamiana* leaves were co-infected with 0.1 µg BaMV-S transcripts [36] and 0.1 µg BSF4 satBaMV RNA transcripts [19] obtained by T7 RNA polymerase (Promega) transcription, then harvested at 7 days post-infection (dpi).

### RNA isolation, RT-PCR cloning, and sequencing

Total RNA was extracted from leaves as described [37] and was used as a template for RT-PCR reaction. The polyadenylation primer Tn836 (5′-CGTACCGAATTCT_15_-3′, *Eco*RI site underlined) was used for first-strand cDNA synthesis with SuperScriptIII one-step RT-PCR system (Invitrogen), followed by PCR amplification with the high-fidelity Platinum *Taq* DNA polymerase (Invitrogen) and primer pairs for BS-5′ (5′-GAAAACTCACCG-3′) and Tn836. The PCR product was then *Eco*RI-digested, gel-purified, and cloned into the *Stu*I/*Eco*RI sites of the pCass2 vector [38]. Plasmids were purified and used as templates for DNA sequencing to determine the identity of satBaMV isolates.

### Sequence analyses

Clustal Omega [39] from EMBL-EBI (http://www.ebi.ac.uk/Tools/msa/clustalo/) was used to align satBaMV sequences, with manual adjustment. The sequence alignment file is available upon request. Several recombination detection algorithms, such as RDP [40], GENECONV [41], Bootscan [42], MaxChi [43], Chimaera [44], SiScan [45], and 3Seq [46], all implemented in RDP4 beta 4.24 [47], were used to detect recombination among satBaMV sequences. MEGA 5.2.1 [48] was used to estimate nucleotide diversity and conduct selection tests. To determine the standard error associated with the estimated nucleotide diversity, 500 bootstraps were conducted. The online service, Intrapop neutrality tests (http://wwwabi.snv.jussieu.fr/achaz/neutralitytest.html), was used for Tajima’s *D* [49] and Fu and Li’s D_2_* and F* [50] tests. MrBayes 3.2 [51] was used to reconstruct phylogenetic relationships. Instructions in the command block was generated with the web service by R. Page and S. R. Santos (https://131.204.120.103/srsantos/mrbayes_form/index.html) and modified to fit our dataset. GTR (General Time Reversible) was used for the nucleotide substitution model and gamma-distribution the variable rate model. In this study, 1,000,000 generations were generated and 25% burn-in was used for each run. The resulting consensus trees were visualized by FigTree 1.3.1 [52].

## Results

We collected seven leaf samples – six from various bamboo species from locations in Taiwan, China, and India and one from inoculated *Nicotiana benthamiana*, a commonly used tobacco plant in plant virus study, in the greenhouse (Figure 1) – to survey the population structures of satBaMVs. The satBaMV genomic sequences were determined by sequencing cloned RT-PCR products. A total of 728 cDNA clones were sequenced, with about 60 to 120 clones from each sample, although not all clones contained the satBaMV sequences (Table 1). Unexpectedly, more clones from the field samples did not contain the satBaMV sequences (13–34%) as compared with the greenhouse sample (∼4%) (Table 1). After removing failed clones, 595 satBaMV-containing sequences were used for further analyses.

**Table 1:** Cloning effort and genome length distribution of satBaMV.

Population*		Bo	Bv	Bvu	Dl	DlM	BvuI	B+F4
		cDNA cloning effort						
	Success	69	102	87	77	52	99	109
	Fail	30	16	19	40	9	15	4
	Fail rate	0.303	0.136	0.179	0.342	0.148	0.132	0.035
		**Percentage of genome length**						
Genome size†	Full-length	78.3	84.3	90.8	66.2	67.3	98.0	86.2
	Short 1	21.7	6.9	9.2	1.3	15.4	1.0	13.8
	Short 2				10.4	1.9	1.0	
	Short 3		1.0			1.9		
	Short 4				5.2			
	Short 5		7.8		9.1	13.5		
	Short 6				3.9			
	Short 7				3.9			

*For population designation, see Figure 1 legend.

† Genome size in nucleotides. Full-length: 799–842; Short 1: 693–733; Short 2: 612–661; Short 3: 556; Short 4: 473–478; Short 5: 397–408; Short 6: 324–357; Short 7: 173–221.

### Baseline error rate from RT-PCR

To establish the baseline error rate introduced by RT-PCR, we used a plasmid-borne satBaMV cDNA as the template to generate RNA transcripts with the T7 RNA polymerase, followed by reverse transcription and PCR amplification under the same conditions used for field samples. We sequenced 18 clones and found only 4 substitution polymorphic sites, all singletons, in 17 full-length sequences of 836 nt and one length polymorphism of 732 nt (with deletion from nucleotides 189 to 292, based on the BSF4 sequence in GenBank accession no. AY205227). The mean nucleotide diversity [53], a population genetic statistic estimating the average number of nucleotide differences per site between two random sequences in a given population, was estimated to be 0.0006±0.0003.

To detect the presence of recombination in our data, we subjected aligned full-length satBaMV sequences to various recombination-detection algorithms (see Materials and Methods). Except for 2 sequences (L-89 and L-104), both from *D. latiflorus* Munro at the Taipei Botanical Garden, we found no evidence of recombination in our samples.

Overall, our results confirmed that RT-PCR introduces a small amount of sequence diversity in a given population study.

### Apparent genome length polymorphism

Of the 595 sequences we analyzed, 496 more or less contained the full-length satBaMV sequences, with genome sizes ranging from 799 to 842 nt. Most of the genomes (69%) in the “full-length” category were 836 nt. The remaining 99 sequences all have deletions to various degrees and were classified into 7 categories based on genome length (Table 1). The group of 693–733 nt (“Short 1” category) was the second most common genome size found in all populations, except for populations from *D. latiflorus* Munro at the Taipei Botanical Garden (Dl) and *B. vulgaris* from India (BvuI). Both populations had ∼1% genomes in the “Short 1” category, whereas others had 7% to 22% sequences belonging to this category. SatBaMVs isolated from both *D. latiflorus* Munro (Dl and DlM) hosts had a wider range of genome sizes, down to approximately 173–221 nt (“Short 7” category in Table 1).

As shown above, the 732-nt variant is likely a laboratory RT-PCR artifact. For the sequences in the 731–733-nt range, all had exactly the same deletion. Furthermore, individuals from a shorter category (*e.g.*, “Short 3”) may be derived from a longer category (*e.g.*, “Short 1”) (see Figure S1). Because of this uncertainty, we removed all deletion mutants for subsequent analysis.

### Levels of genetic diversity among satBaMV populations

**Figure 2:**
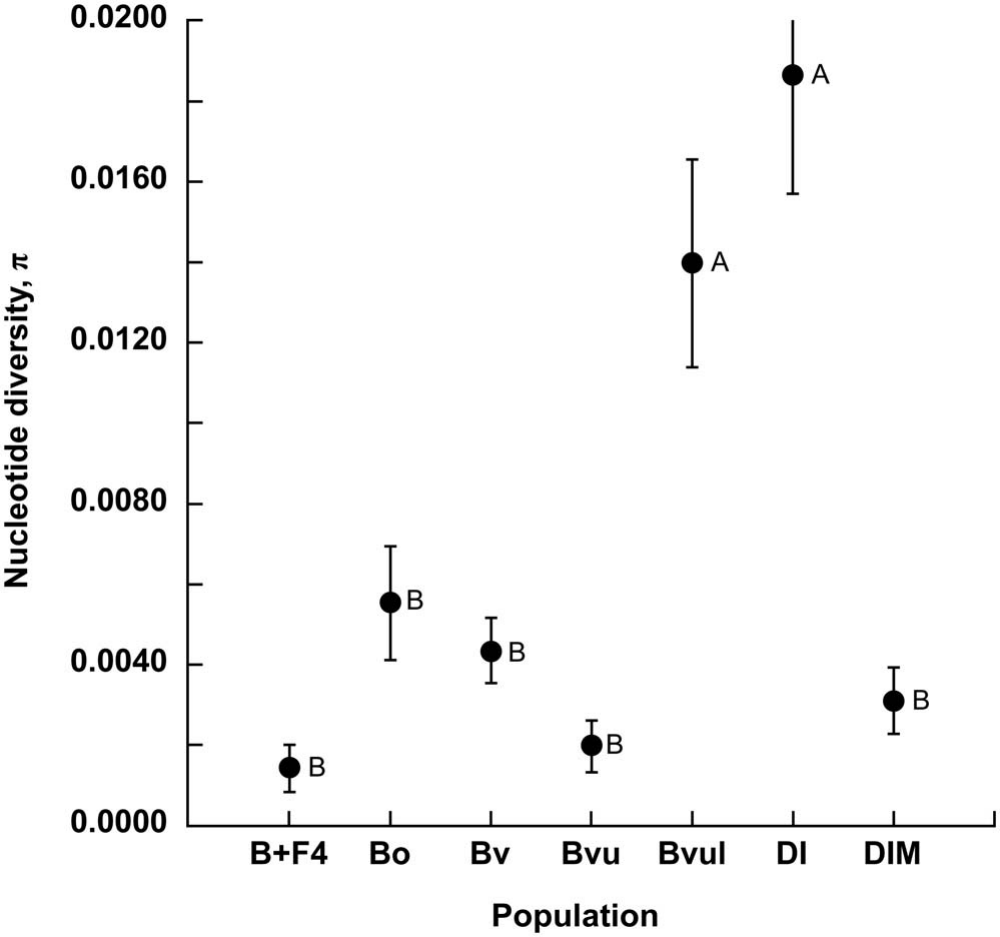
Nucleotide diversity of satBaMV populations. MEGA 5.2.1 [48] was used to estimate the nucleotide diversities and associated standard errors (vertical bars) of various satBaMV populations. Letters denote statistically different groups based on unplanned multiple comparisons with the Tukey-Kramer method [54]. For population designation, see Figure 1. B+F4 denotes the population from greenhouse-inoculated *N. benthamiana.*

We adopted nucleotide diversity π [53] to measure the genetic diversity of satBaMV populations. The mean estimated nucleotide diversity ranged from 0.0014±0.0006 (the greenhouse-inoculated *N. benthamiana*) to 0.0187±0.0030 (*D. latiflorus* Munro, Dl), approximately a 13-fold difference (Figure 2). Among the field samples, the lowest nucleotide diversity was with *B. vulgaris* Schrader ex Wendland (Bvu), with a value of 0.0020±0.0006.

To test whether these estimated values significantly differed, we used the Tukey-Kramer method for unplanned multiple comparisons [54] (Box 9.7, Part IV). SatBaMV populations from *D. latiflorus* Munro (DI) and *B. vulgaris* Schrader ex Wendland in Inida (Bvul) had the highest nucleotide diversity, and the rest had a lower value (Figure 2). Among our leaf samples were several contrasting satBaMV populations, derived from the same bamboo species but different locations or from the same location but different species. For example, for *B. vulgaris* Schrader ex Wendland, we had one sample from the Taipei Botanical Garden and one from India (Bvu vs. BvuI). As well, for *D. latiflorus* Munro, we had one sample from the Taipei Botanical Garden and one from Pingtung (Dl vs. DlM). We also had satBaMV populations from *B. vulgaris* or *D. latiflorus* at the Taipei Botanical Garden (Bvu vs. Dl). Interestingly, all pairwise comparisons revealed significant differences in nucleotide diversity (Figure 2).

Our analysis showed that nucleotide diversity among satBaMV populations varied greatly, indicating the presence of different demographic and/or evolutionary histories among these satBaMV populations. Furthermore, neither host species nor geographic location predominantly determined the level of nucleotide diversity within a given satBaMV population.

### Phylogeny of the satBaMVs

**Figure 3:**
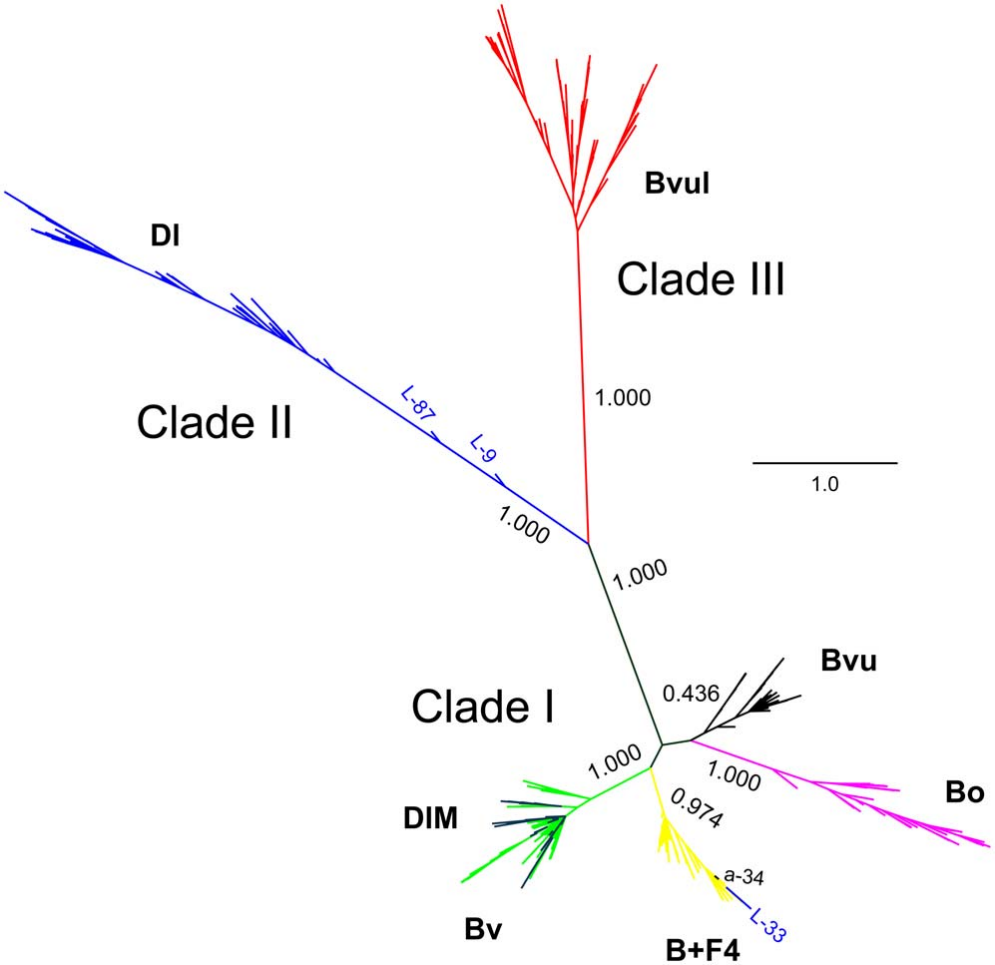
Phylogenetic relationship among different satBaMV populations. Unrooted Bayesian tree was constructed with full-length satBaMV genomic sequences. For clarity of presentation, sequence names are not shown, except for the listed (see text for details). Yellow, B+F4; magenta, Bo; green, Bv; black, Bvu; red, BvuI; blue, Dl; purple, DlM. Posterior probabilities are shown for major branches.

The mean within-population nucleotide diversity was estimated at 0.0080±0.0012 (or 0.0071±0.0009 with the greenhouse sample), and the mean between-population diversity 0.0430±0.0066 (or 0.0391±0.0056 with the greenhouse sample), approximately a five-fold difference. This result, together with previous findings [23], suggests that each satBaMV population evolves independently, thus accumulating sequence divergence across all populations. This interpretation is further corroborated by the observation that most satBaMV sequences clustered with members from the same population, forming three well-supported clades (Figure 3). Clade I is consisted of all satBaMV populations, except most of those isolated from *D. latiflorus* Munro at the Taipei Botanical Garden (Dl) and all of those from *B. vulgaris* Schrader ex Wendland in India (BvuI). Clade II is consisted of sequences found exclusively in *D. latiflorus* Munro (Dl) at the Taipei Botanical Garden and Clade III sequences exclusively from *B. vulgaris* Schrader ex Wendland in India (BvuI). These three clades also corresponded with the levels of nucleotide diversity shown above, with all populations in Clade I having lower nucleotide diversity and both Clades II and III having significantly higher nucleotide diversity.

Most individual sequences tended to cluster with individuals from the same population (Figure 3), with some exceptions. The most notable exception was the well-supported clustering of sequences from *D. latiflorus* Munro cv. “Mei-nung” (DlM) in southern Taiwan and those from *B. ventricosa* McClure (Bv) in Hainan Island, China (Figure 3). This clustering suggests a recent migration event(s) of satBaMV between these two geographic locations. The second exception is the clustering of a single sequence from *B. vulgaris* (isolate a-34) and from *D. latiflorus* (isolate L-33), both from the Taipei Botanical Garden, with members from the greenhouse *N. benthamiana*. This second exception may not be too surprising, because the BSF4 isolate used to inoculate greenhouse *N. benthamiana* was originally isolated from *B. vulgaris* at the Taipei Botanical Garden in 1994 [17].

### Selection of satBaMV P20 function

Among our satBaMV populations, the proportion of single nucleotide polymorphism (nucleotide substitutions that appear only once in the sample population) was relatively high, from 58% (Bo) to 79% (DlM) or to 88% when including the greenhouse (B+F4) population. The finding of the presence of excess rare variants is further corroborated with a more rigorous analysis with the Tajima’s *D* test [49], a statistical test designed to distinguish between neutral evolution of nucleotide sequences from non-neutral mechanisms, such as natural selection. All populations had significant negative *D* values, indicating the presence of excess rare variants (Table 2). Similar tests, such as the Fu and Li’s D* and F* [50] tests, showed the same pattern (data not shown).

**Table 2:** Population genetic statistics and tests of satBaMV populations.

Population*	Bo	Bv	Bvu	Dl	DlM	BvuI	B+F4
Tajima’s *D* (*p*)†	−1.508 (0.042)	−2.629 (<0.001)	−2.230 (<0.001)	−1.530 (0.037)	−1.981 (0.007)	−1.466 (0.041)	−2.268 (<0.001)
*Z* (*p*)‡	1.278 (0.102)	1.189 (0.118)	2.094 (0.019)	2.315 (0.012)	2.094 (0.019)	2.474 (0.007)	0.284 (0.389)

*For population designation, see Figure 1 legend.

† Tajima’s *D* test and associated probability in bracket. For Tajima’s *D* test, *H*_A_ = *D*_obs_>*D*_exp,_ with 100,000 replicates.

‡ *Z* test and associated probability in bracket. *Z* = *d*S*–d*N and the *H*_A_ = *d*N<*d*S, with 500 replicates.

Typically, population expansion and purifying selection are the two mechanisms commonly invoked to explain the finding of a negative Tajima’s *D* value [49], although these two explanations are not mutually exclusive. In our case, the sole protein-encoding gene *P20* in the satBaMV genome provided us the opportunity to test whether natural selection for P20 function plays a role in the evolution of the satBaMV sequence. One of the commonly used methods for detecting the signatures of natural selection is the *d*N/*d*S test [55]. If the ratio (ω) is <1, then it is customarily seen as evidence of purifying selection. However, if ω>1, then it is indicative of positive selection favoring amino acid substitution. If ω = 1, then neither purifying selection nor positive selection is responsible for the observed nucleotide sequence pattern. With the commonly used *d*N/*d*S test implemented in MEGA [48], we found that 4 of the 7 populations (*i.e.*, Bvu, Dl, DlM, and BvuI) showed significantly smaller *d*N values than corresponding *dS* values (Table 2), indicating the presence of purifying selection. Not all populations had significant differences between *d*S and *d*N values (*e.g.*, B+F4, Bo, and Bv), but all had a higher *d*S than *d*N value (Table 2). Together with the finding of negative Tajima’s *D* values, these results suggest a consistent explanation that purifying selection against less-fit P20 variants are at least in part responsible for the evolution of satBaMV.

## Discussion

### Genome-size polymorphism and RT-PCR artifact

RT-PCR cloning followed by DNA sequencing is commonly used for population genetic studies of RNA viruses. However, the very act of RT-PCR can introduce sequence artifacts in the form of point mutations, deletion/insertion [56], [57], and sometimes recombination [58]. To minimize laboratory artifacts, we used a high-fidelity DNA polymerase for our amplifications. Nevertheless, we could not prevent the occurrence of deletion mutations in our samples, specifically the 732-nt variant, and possibly others as well. The deletion is possibly due to a secondary structure in the satBaMV genome encountered by the reverse transcriptase during the first-strand cDNA synthesis [57]. While the 732-nt variant may be an RT-PCR artifact, the phylogenetic relationship within this group still mirrored that of its full-length counterpart. For example, individual sequences from the same population still clustered together, with the same exception for those isolated from *D. latiflorus* Munro cv. “Mei-nung” in southern Taiwan and those from *B. ventricosa* McClure in Hainan Island, China, which formed a well-supported clade (data not shown). Furthermore, within each satBaMV population, the 732-nt sequences were interspersed among the full-length ones, which suggests rather than originating from a single or a few founding mutational events, most of the 732-nt sequences represent independent deletion events (data not shown). One possible explanation is that within a given satBaMV population, a portion of the genomes assumes a stable alternative secondary structure that, when encountered by a reverse transcriptase during RT-PCR, would facilitate the creation of a deletion mutation.

The 732-nt variant was demonstrated to be a laboratory artifact, but the remaining shorter genomes, especially those in both of the *D. latiflorus* Munro hosts (Table 1), may have a more biologically relevant origin. An *in planta* study with a satRNA associated with CMV (satCMV) [59] showed that the deletion mutation rate of the helper viral replicase depended on the plant host. The deletion rate was approximately 7-fold higher in pepper than tobacco. A similar dependency of the BaMV replicase on bamboo species could explain an increased proportion of the satBaMV genomes deleted when in *D. latiflorus* Munro.

### Phylogenetic relationships among satBaMV populations

**Figure 4:**
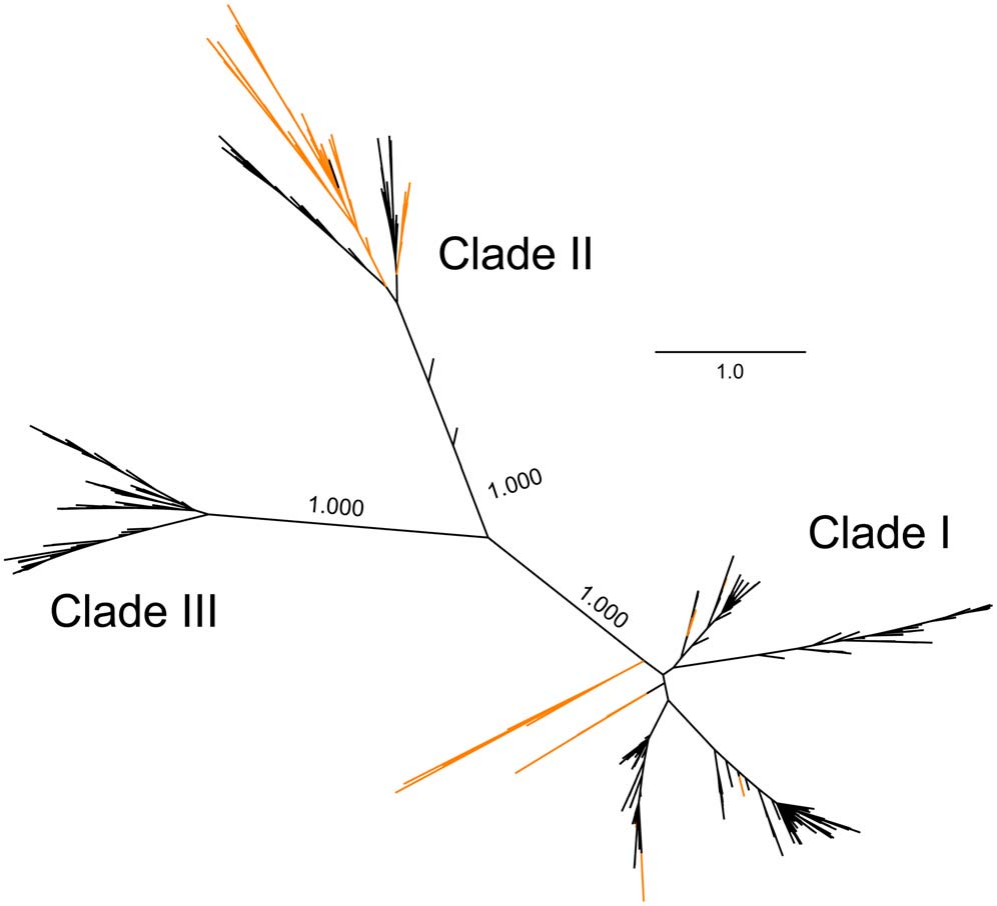
Phylogenetic relationship among all known full-length satBaMV. Unrooted Bayesian tree was constructed using full-length satBaMV genomic sequences from the current (black) and previous (orange) studies. Previous sequences used are from GenBank accession numbers AY205159–AY205231, except AY205176, AY205199, and AY205209. Posterior probabilities are shown for major branches.

Our previous surveys of satBaMV in Taiwan revealed two readily discernible clades: one with only satBaMVs isolated from *D. latiflorus* Munro (but see below) and the other including all other samples, as well as some from *D. latiflorus* Munro [23], [29]. We revealed a third exclusive clade composed of only individuals from *B. vulgaris* in India (BvuI) (Figure 3). When previous and current full-length satBaMV sequences were used in phylogenetic reconstruction, the same three main clades were retrieved (Figure 4). Because collection of these samples spanned more than a decade, the existence of these distinct clades suggests that they are quite stable in the satBaMV populations.

**Figure 5:**
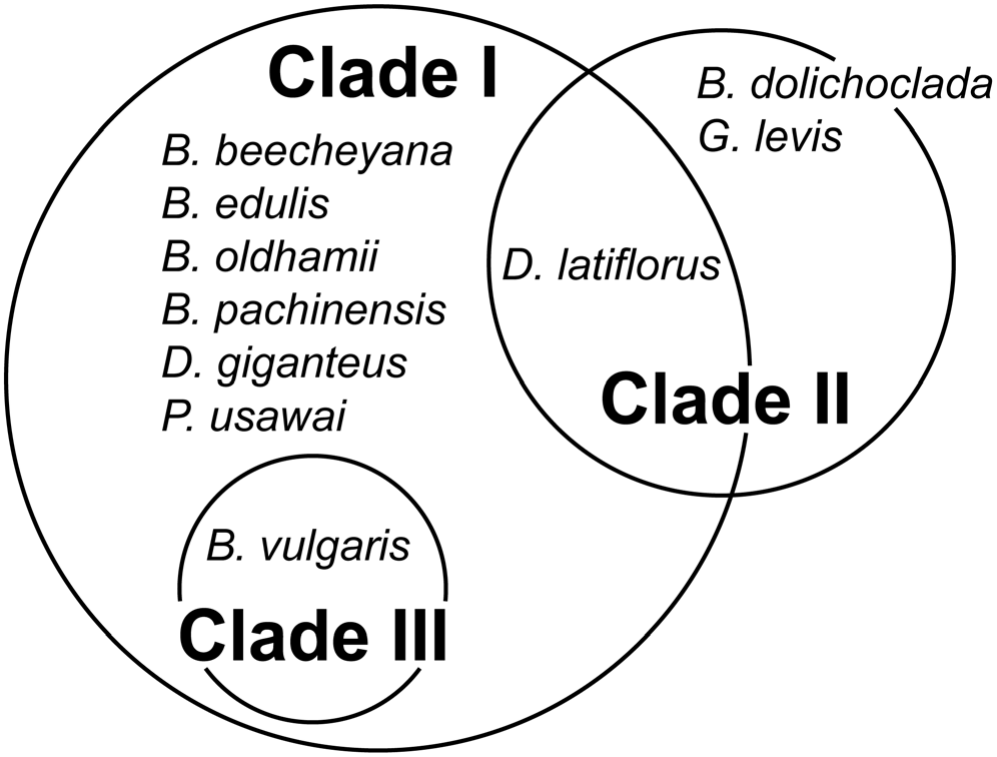
Distribution of bamboo species infected with different satBaMV clades. Information from both current and previous studies [23], [29] were used to group bamboo species according to the satBaMV clade(s) found infecting them.

Clade II was composed of almost entirely individuals isolated from *D. latiflorus* Munro from various locations in Taiwan. Only two other bamboo species, *B. dolichoclada cv.* Stripe [29] and *G. levis* [23], were found to carry satBaMVs in this clade. However, at least eight different bamboo species were found infected with Clade I in Taiwan (Figure 5). Whether the paucity of host species for Clade II is due to biased sampling or genuine restricted host range is not known. Nevertheless, Clade II seems to have a narrower host range but approximately 7-fold higher genetic diversity (Figure 2), than Clade I. Clade I may have initially derived from the high-diversity Clade II, with subsequent host range expansion. Alternatively, Clade II may have derived from Clade I, accompanied by an increased mutation rate of the helper BaMV replicase in a different host, as occurred with the satCMV [59], thus maintaining a high steady-state nucleotide diversity in the population. So far, we cannot differentiate between these 2 scenarios. As for Clade III, which is only found in India with *B. vulgaris*, our current sampling is too limited to determine whether this clade would have a narrow host range or not.

**Figure 6:**
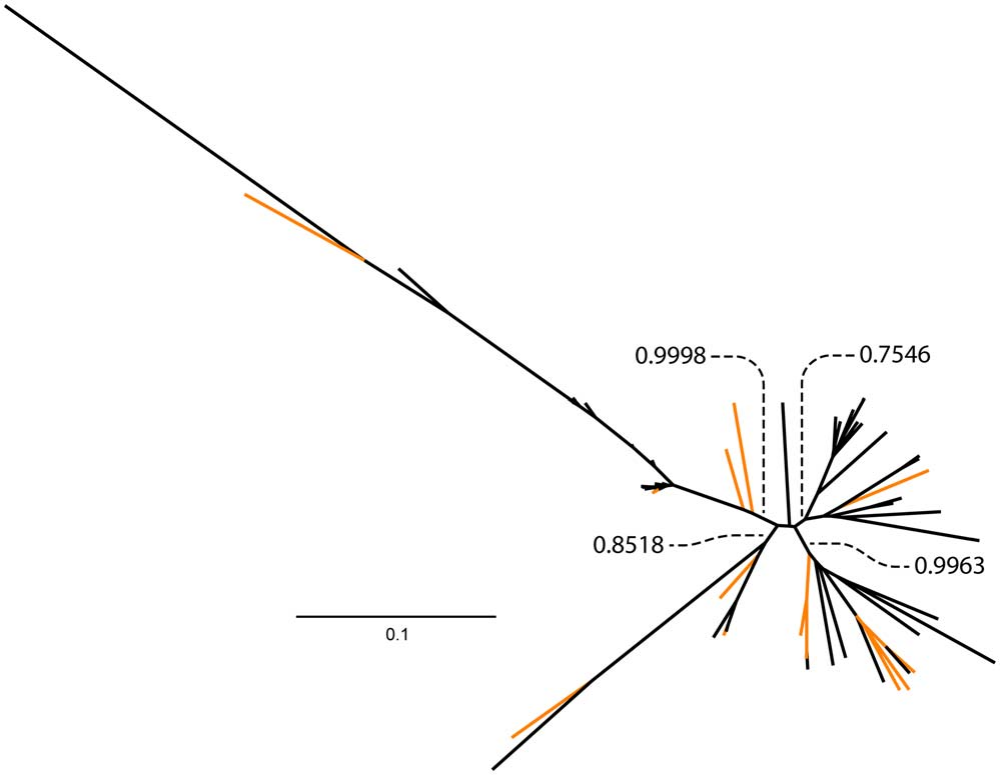
Phylogenetic relationship among BaMV isolates. Unrooted Bayesian tree was constructed using sequences encoding the RNA-dependent RNA polymerase from BaMV isolated from various bamboo species. Highlighted isolates (orange) denote BaMVs from the Ma bamboo (*D. latiflorus* Munro). Posterior probabilities (dashed lines) are shown for major branches.

The existence of distinct satBaMV clades prompted us to examine the phylogenetic relationship of the helper BaMV isolates. Our preliminary analysis showed no immediately discernible clustering among the BaMV isolates (Figure 6), as compared with what is found for satBaMV. This preliminary result suggests that satRNAs, while obligatorily requiring the helper viral functions for replication, encapsidation, and efficient movement, could have very different evolutionary histories from their cognate helper viruses.

### Evolution of interference phenotype

Two prototypical isolates, BSL6 [29] and BSF4 [17], are commonly used in studies of satBaMV. The former interferes greatly with the replication of the helper BaMV [30], thus reducing the severity of disease symptoms, whereas the latter interferes only slightly, thus showing a typical mosaic symptom on co-infection with BaMV [19], [30]. Competition for access of the BaMV-encoded replicase is thought to be the basis for replication interference [28], [60]. One important determinant for interference is located at the AHSL at the 5′ UTR, of which both the helper BaMV and satBaMV share a similar secondary structure [23], [24]. Within the 5′ AHSL, two nucleotide positions, 60 and 82 (based on the BSF4 sequence [17]), are critical for determining the interference phenotype [28]. If either position contains a U instead of C, the resulting satBaMV would not be able to (or only slightly) interfere with BaMV replication [28]. In our satBaMV populations, all sequences have a C at position 60 (or homologous positions), and almost all have a C at position 82 as well. The only exceptions are the satBaMV population from the greenhouse-infected *N. benthamiana*, for which 38.3% have a U in position 82, and one sequence (isolate a-34) from the *B. vulgaris* and three sequences (isolates L-9, L-33, and L-87) from *D. latiflorus* Munro that also have a U at the homologous position 82. The original sequence used for infecting *N. benthamiana* is BSF4, which contains a U at position 82 [17]. Therefore, even within a short time (*i.e.*, 7 dpi), we observed the evolution of U to C at position 82 from the initial 100% to 38.3%. Presumably, selection for accessing the replicase, against both the ancestral BSF4 and the helper BaMV, drives the evolution at this position. Interestingly, both sequences a-34 and L-33 are found clustered with the satBaMV population from the greenhouse-infected *N. benthamiana* (Figure 2), suggesting that satBaMV sequences very similar to BSF4, first reported in 1994 [17], still exist in the field and coexist with other satBaMV clades. Also, all sequences isolated from *D latiflorus* Munro (Dl) (except the L-33) clustered together, forming a well-supported Clade II, but both L-9 and L-87 seem to be distantly related to all the other sequences in this clade (Figure 3). These two sequences may represent an intermediate stage between Clade I and II satBaMVs.

With our current phylogeny, BSF4 belongs to Clade I and BSL6 to Clade II. However, mapping of the nucleotide state at positions 60 and 82 showed that, in the field, both clades are dominated by sequences that may greatly interfere with BaMV replication. The same pattern was also found with individuals in Clade III. Most of the satBaMV sequences found in the field probably also have a C in positions that are homologous to position 82. Other features of the genome, such as the AHSL structure and the surrounding sequence in the 5′ UTR region, could influence the interference phenotype, but the evolution of these satBaMV clades is unlikely to be driven by the evolution of the interference phenotype.

### Selection of the P20 protein

The satBaMV-encoded P20 protein is important in long-distance movement through the phloem [17], [21], [22]. P20 plays a supporting role in satBaMV replication as well. For example, none of the truncated P20 mutants could reach the same accumulation level as that of the wild-type P20 in the protoplast assay [19]. Furthermore, other satRNA-encoded proteins are required for satRNA replication [61]–[63]. Therefore, P20 likely facilitates its replication as well as long-distance movement within the infected host.

Our analysis suggests that the *P20* gene is under purifying selection against less-fit P20 proteins, which, presumably, are not efficient in facilitating satBaMV movement within the host plant and/or its own replication. However, for purifying selection to be the main driving force for the evolution of satBaMV, the interaction between individual satBaMV genomes and the P20 proteins inside an infected cell needs to be predominantly *cis* in nature; that is, the genome that sustained a deleterious mutation in the *P20* gene should, on average, “pay” a fitness cost for expressing a less fit P20 protein, either as a result of less-efficient long-distance and/or less-efficient replication of the satBaMV genome. However, if P20 is a diffusible protein that interacts with any satBaMV genomes for movement and/or replication (*i.e.*, a mainly *trans*-acting interaction), then other fitter P20 proteins can still facilitate the movement and/or replication of the satBaMV genome that carries a less-fit *P20* mutation. The within-cell complementation of fit P20 proteins with mutant satBaMV genomes could greatly reduce or completely abolish the effect of purifying selection.

The replication of the potexviruses, of which BaMV is a member, may provide clues to whether the prerequisite *cis* interaction is possible. Replication of many positive-strand plant RNA viruses is accompanied by extensive intracellular membrane reorganization, usually involving the formation of viral-induced membrane vesicles or invagination [64]. One consequence of RNA genome compartmentalization is the almost exclusive interaction between the protein products and the genomes expressing them, effectively a *cis* interaction. Whether BaMV replication, and by extension satBaMV replication, also involves rearranged membrane structures is unclear. Although other potexviruses [65] adopt such a strategy for genome replication. In the case of satBaMV, it would be interesting to know if co-infection of a wild-type satBaMV with a P20 mutant, such as the one with the P20 gene replaced by *cat* [19], may result in the translocation of the mutant satBaMV, thus establishing the *cis*-acting nature between P20 and the genome expressing it.

## Supporting Information

Figure S1 **Genomic deletions in satBaMV isolates.** The prototypical satBaMV genome of the BSF4 isolate (GenBank accession no. AY205227) was used to illustrate region(s) of deletion in the genomes of the most common isolate in each “short” category listed in Table 1. Open bar shows the encoded *P20* gene and hatched bars the regions of deleted genome. Numbers with vertical bars indicate the nucleotide positions (of BSF4) for the start and end of the *P20* gene or the deleted regions. Numbers at the end of each genome show the specific genome length. Various single-nucleotide deletions, found in some genomes, are not shown in the illustration.
(DOCX)
