# Supplementary figures and images for: Genetic Diversity and Evolution of Satellite RNAs Associated with the Bamboo Mosaic Virus
Source: PLoS One. 2014 Oct 2;9(10):e108015. doi: 10.1371/journal.pone.0108015 (PMC4183488; doi:10.1371/journal.pone.0108015)

**Fig. S1**


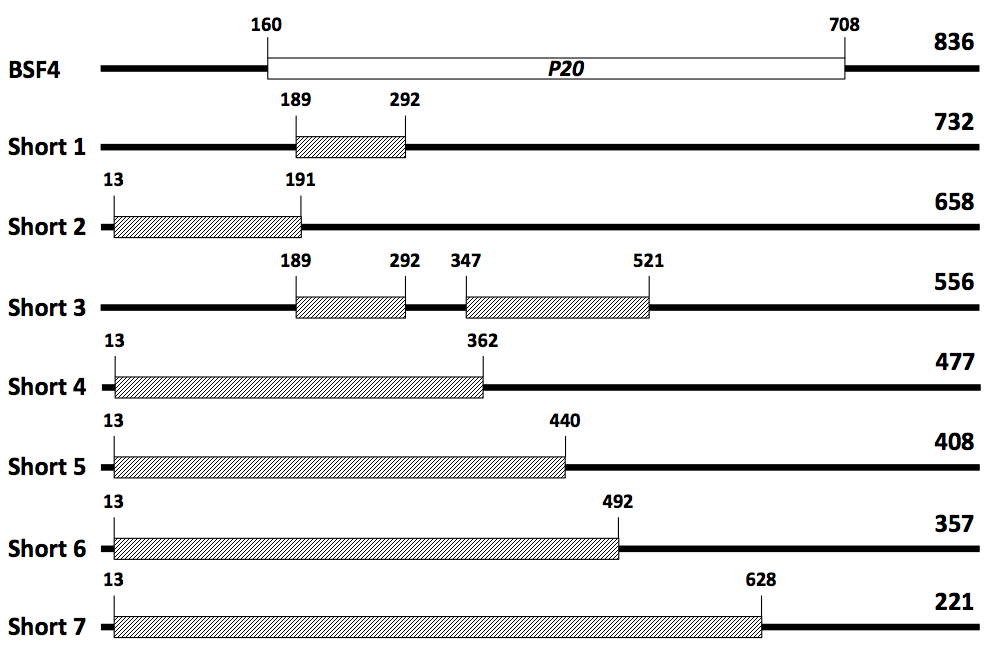

Supplement: Figure S1 — Genomic deletions in satBaMV isolates. The prototypical satBaMV genome of the BSF4 isolate (GenBank accession no. AY205227) was used to illustrate region(s) of deletion in the genomes of the most common isolate in each “short” category listed in Table 1. Open bar shows the encoded P20 gene and hatched bars the regions of deleted genome. Numbers with vertical bars indicate the nucleotide positions (of BSF4) for the start and end of the P20 gene or the deleted regions. Numbers at the end of each genome show the specific genome length. Various single-nucleotide deletions, found in some genomes, are not shown in the illustration. (DOCX) [file pone.0108015.s001.docx]
